# Dietary Sources of Methylated Arsenic Species in Urine of the United States Population, NHANES 2003–2010

**DOI:** 10.1371/journal.pone.0108098

**Published:** 2014-09-24

**Authors:** B. Rey deCastro, Kathleen L. Caldwell, Robert L. Jones, Benjamin C. Blount, Yi Pan, Cynthia Ward, Mary E. Mortensen

**Affiliations:** Centers for Disease Control and Prevention, National Center for Environmental Health, Division of Laboratory Sciences, Atlanta, Georgia, United States of America; Institute for Health & the Environment, United States of America

## Abstract

**Background:**

Arsenic is an ubiquitous element linked to carcinogenicity, neurotoxicity, as well as adverse respiratory, gastrointestinal, hepatic, and dermal health effects.

**Objective:**

Identify dietary sources of speciated arsenic: monomethylarsonic acid (MMA), and dimethylarsinic acid (DMA).

**Methods:**

Age-stratified, sample-weighted regression of NHANES (National Health and Nutrition Examination Survey) 2003–2010 data (∼8,300 participants ≥6 years old) characterized the association between urinary arsenic species and the additional mass consumed of USDA-standardized food groups (24-hour dietary recall data), controlling for potential confounders.

**Results:**

For all arsenic species, the rank-order of age strata for median urinary molar concentration was children 6–11 years > adults 20–84 years > adolescents 12–19 years, and for all age strata, the rank-order was DMA > MMA. Median urinary molar concentrations of methylated arsenic species ranged from 0.56 to 3.52 µmol/mol creatinine. Statistically significant increases in urinary arsenic species were associated with increased consumption of: fish (DMA); fruits (DMA, MMA); grain products (DMA, MMA); legumes, nuts, seeds (DMA); meat, poultry (DMA); rice (DMA, MMA); rice cakes/crackers (DMA, MMA); and sugars, sweets, beverages (MMA). And, for adults, rice beverage/milk (DMA, MMA). In addition, based on US (United States) median and 90th percentile consumption rates of each food group, exposure from the following food groups was highlighted: fish; fruits; grain products; legumes, nuts, seeds; meat, poultry; and sugars, sweets, beverages.

**Conclusions:**

In a nationally representative sample of the US civilian, noninstitutionalized population, fish (adults), rice (children), and rice cakes/crackers (adolescents) had the largest associations with urinary DMA. For MMA, rice beverage/milk (adults) and rice cakes/crackers (children, adolescents) had the largest associations.

## Introduction

Arsenic is an ubiquitous metalloid element present in the environment as inorganic species with different oxidation states, or as part of organic compounds. Inorganic arsenic species of greatest environmental health concern have oxidation states of +3 (As(III) or arsenite) or +5 (As(V) or arsenate), and are classified as human carcinogens [1], [2]. People with chronic exposure to high levels of inorganic arsenic species in drinking water have manifested neurotoxicity, skin lesions, gastrointestinal and liver dysfunction, and cardiovascular disease [3]–[6]. In the United States, regulations limit arsenic in public water systems and in bottled water to 10 µg/L [7], [8]. The methylated arsenic species dimethylarsinic acid (DMA) and monomethylarsonic acid (MMA) have both been detected in soils, fresh and marine waters, and fish and shellfish.

Human metabolic biotransformation of inorganic arsenic involves reduction from As(V) to As(III), followed by oxidative methylation to monomethyarsinic acid (MMA) and dimethylarsinic acid (DMA) [5], [9]. Since most arsenic species absorbed from the gastrointestinal tract is excreted in urine within 1–3 days [10]–[15], urinary arsenic is well-suited as an exposure biomarker to correlate with NHANES 24-hour dietary recall data. Although inorganic species have been a focus of concern because of their carcinogenicity [16], certain methylated arsenic species may be carcinogenic [17], [18]. Indeed, there is greater awareness that methylation of inorganic arsenic species may also affect susceptibility to arsenic-induced disease and may vary among individuals depending on genetic polymorphism, dose, age, selenium intake, as well as folate and homocysteine status [5], [17], [19]–[27]. Exposure to DMA [28] and MMA [29]–[35] have produced tumors in animal studies, resulting in concerns about human carcinogenicity.

Several studies have examined selected US (United States) foods for total and inorganic arsenic species content [36]–[40], or have examined the relationship between consumption of selected foods and total urinary arsenic [36], [39] and speciated arsenic [41]–[43]. Using US FDA's (Food and Drug Administration) Total Diet Study 1991–1996, Tao and Bolger [44] reported that total arsenic concentration in food was highest in seafood, followed by rice/rice cereal, mushrooms, and poultry. We analyzed NHANES (National Health and Nutrition Examination Survey) data from 2003–2010 for associations between increased consumption of a comprehensive set of food groups and urinary concentrations of arsenic species that share human metabolic biotransformation pathways [9]. This approach was successfully utilized for a similar assessment of dietary exposure to perchlorate in NHANES 2001–2008 data [45].

## Materials and Methods

### Study Design

NHANES, conducted by the National Center for Health Statistics (NCHS) of the Centers for Disease Control and Prevention (CDC), collects serial cross-sectional data from a complex, multistage probability sample representative of the civilian, non-institutionalized population of the United States. The data used in this study are publicly available for download at http://www.cdc.gov/nchs/nhanes.htm. This website also provides details on the study design and recommended statistical techniques for analyzing NHANES data. Food consumption information was collected from participants using structured questionnaires administered by trained interviewers. Urinary arsenic species were measured in a single spot urine sample obtained from a one-third subsample of participants 6 years and older who provided spot urine specimens during physical examinations in the NHANES 2003–2010 cycles. Urine was collected and stored in arsenic-free containers and shipped on dry ice to CDC's National Center for Environmental Health laboratory, where samples were stored at or below −20°C until analyzed and typically analyzed within three weeks of collection (total urinary arsenic concentrations are stable at approximately −70°C for up to three years [46]). Arsenic species in urine were separated by high performance liquid chromatography, then directed to an ELAN DRC II ICP-MS (PerkinElmer SCIEX, Concord, Ontario, Canada) [47]. The limits of detection (LOD) were: As (III) 1.2 µg/L; As (V) 1.0 µg/L; DMA 1.7 µg/L; and MMA 0.9 µg/L [47], [48]. Measurements below the LOD were substituted with the quotient of the LOD divided by the square-root of two. Urine creatinine measurements in NHANES 2003–2006 were made using a CX3 Analyzer that employed the Jaffe reaction [49]; in NHANES 2007–2010 these measurements were made using a Roche/Hitachi Modular P Chemistry analyzer that employed an enzymatic method using creatinase [50]. Serum folate measurements in NHANES 2003–2006 were made using the BioRad Quantaphase II Radioassay [51]; in NHANES 2007–2010 these measurements were made using a microbiologic assay [52], [53]. Reported measurements met the accuracy and precision specifications of the quality-control/quality-assurance program of the CDC National Center for Environmental Health, Division of Laboratory Sciences [54].

### Statistical Analysis

NHANES is a complex survey where participants are recruited through a multistage sampling design that involves stratification, clustering, and oversampling of specific population subgroups. It is therefore necessary to account for this complex design in order to properly estimate variances of regression coefficients and to produce unbiased, nationally representative statistics [55]. Robust estimation may be accomplished through Taylor series linearization and applying survey sample weights to each survey participant. We used this estimation approach as it was implemented in the DESCRIPT and REGRESS subroutines of SUDAAN version 11.0.0 [56] called from the SAS version 9.3 statistical software application [57]. We fit a set of sample-weighted, age-stratified linear regression models to publicly available NHANES data from the 2003–2010 survey cycles [58] where the dependent variable was the urinary molar concentration of one of four arsenic species [nmol/L]: As(III) (arsenous acid), As(V) (arsenic acid), DMA (dimethylarsinic acid), and MMA (monomethylarsonic acid). Using molar units enables concentrations to be directly compared between arsenic species. We refer to As(III) and As(V) as “inorganic” arsenic species, and DMA and MMA as “methylated” arsenic species. Since urinary concentrations can be influenced by urine dilution, which can vary markedly throughout the day within each individual [59], we accounted for urinary dilution by normalizing urinary arsenic molar concentrations to urinary molar creatinine (Cre) for the summary statistics in Tables 1–3 [µmol/mol Cre]. For the same reason, we accounted for urinary dilution by including urinary creatinine [g/L] as a model predictor in the regression models. In addition, because the distribution of urinary arsenic measurements were highly right-skewed, these data were transformed with the natural log for evaluating the statistical significance of regression slopes and we report the p-values from the ln-transformed regression models. To facilitate interpretability, however, we report slopes and their 95% confidence intervals estimated from identical un-transformed regression models. Statistical significance was set to *p*≤0.05, and marginal significance was set to 0.05<*p*≤0.15.

**Table 1:** Sample-weighted statistics for creatinine-adjusted urinary arsenic species [µmol/mol Cre] in NHANES 2003–2010 data used in the regression models displayed in Tables 3 and 4.

	Median (25 percentile, 75 percentile)		
Arsenic Species	Adults	Adolescents	Children
DMA	2.97 (1.97, 4.81)	2.18 (1.55, 3.18)	3.52 (2.54, 5.36)
MMA	0.66 (0.42, 1.07)	0.56 (0.37, 0.85)	0.78 (0.50, 1.23)

Cre: creatinine.

**Table 2:** Sample-weighted 25^th^, 50^th^, and 75^th^ percentiles for creatinine-adjusted urinary DMA [µmol/mol Cre] for NHANES 2003–2010 data used in the regression models displayed in Tables 3 and 4.

	DMA					
	Adults		Adolescents		Children	
Percent ≥ LOD Used in Analysis		84.2		87.4		86.0
	Sample Size	Median (25%ile, 75%ile)	Sample Size	Median (25%ile, 75%ile)	Sample Size	Median (25%ile, 75%ile)
**Age at Screening [years]**						
6–11					1,044	3.52 (2.54, 5.36)
12–19			1,709	2.18 (1.55, 3.18)		
20–39	1,906	2.65 (1.76, 4.32)				
40–59	1,758	3.07 (2.05, 5.07)				
60–84	1,851	3.30 (2.24, 5.20)				
**Race/Ethnicity**						
Mexican American	1,016	3.35 (2.23, 4.99)	498	2.57 (1.86, 3.67)	326	4.28 (3.08, 5.73)
Non-Hispanic Black	1,091	2.35 (1.58, 3.92)	536	1.87 (1.28, 2.80)	288	3.08 (2.24, 4.51)
Non-Hispanic White	2,819	2.84 (1.94, 4.61)	499	2.05 (1.53, 2.95)	280	3.36 (2.46, 5.03)
Other Hispanic	381	4.39 (2.89, 6.90)	101	3.08 (1.86, 4.49)	81	3.85 (2.80, 5.82)
Other/Multiracial	208	5.18 (3.27, 9.50)	75	3.20 (1.85, 6.67)	69	4.72 (3.15, 8.39)
**Sex**						
Female	2,798	3.28 (2.17, 5.26)	822	2.29 (1.59, 3.25)	524	3.51 (2.49, 5.34)
Male	2,717	2.65 (1.77, 4.28)	887	2.08 (1.52, 3.07)	520	3.53 (2.62, 5.37)

Cre: creatinine.

**Table 3:** Sample-weighted 25^th^, 50^th^, and 75^th^ percentiles for creatinine-adjusted urinary MMA [µmol/mol Cre] for NHANES 2003–2010 data used in the regression models displayed in Tables 3 and 4.

	MMA					
	Adults		Adolescents		Children	
Percent ≥ LOD Used in Analysis		32.9		42.4		29.2
	Sample Size	Median (25%ile, 75%ile)	Sample Size	Median (25%ile, 75%ile)	Sample Size	Median (25%ile, 75%ile)
**Age at Screening [years]**						
6–11					1,044	0.78 (0.50, 1.23)
12–19			1,709	0.56 (0.37, 0.85)		
20–39	1,906	0.60 (0.39, 0.96)				
40–59	1,757	0.66 (0.42, 1.14)				
60–84	1,851	0.74 (0.49, 1.20)				
**Race/Ethnicity**						
Mexican American	1,016	0.66 (0.40, 1.03)	498	0.58 (0.38, 0.85)	326	0.78 (0.54, 1.36)
Non-Hispanic Black	1,091	0.44 (0.32, 0.66)	536	0.45 (0.29, 0.62)	288	0.64 (0.41, 1.03)
Non-Hispanic White	2,818	0.69 (0.45, 1.11)	499	0.58 (0.37, 0.87)	280	0.80 (0.50, 1.28)
Other Hispanic	381	0.73 (0.47, 1.14)	101	0.58 (0.43, 0.86)	81	0.92 (0.58, 1.32)
Other/Multiracial	208	0.89 (0.53, 1.35)	75	0.73 (0.49, 1.24)	69	0.83 (0.54, 1.18)
**Sex**						
Female	2,798	0.75 (0.47, 1.29)	822	0.58 (0.36, 0.91)	524	0.78 (0.49, 1.32)
Male	2,716	0.59 (0.38, 0.90)	887	0.55 (0.37, 0.80)	520	0.78 (0.51, 1.18)

Cre: creatinine.

#### Dietary Consumption by Food Group

Primary interest was in the mass NHANES participants consumed within each USDA (US Department of Agriculture) food group for the 24-hour period (midnight to midnight) preceding the dietary recall interview conducted in-person as part of the physical examination. Data for the 24-hour recall period are contained in the publicly available NHANES Individual Foods – First Day file, which provides a record describing each food, water, or beverage consumed by the participant, including the mass reported consumed and eight-digit USDA food code. Standardized hierarchical food groups can be identified from the USDA code, where the first digit represents one of nine major food groups, and each subsequent digit represents subgroups of increasing specificity [60]. The mass consumed in each food group was summed so that each participant was represented by a single record describing their dietary intake for the previous 24 hours. Each participant's dietary intake was first apportioned over nine food groups: milk products; meat, poultry; eggs; legumes, nuts, seeds; grain products; fruits; vegetables; fats, oils, salad dressings; and sugars, sweets, beverages. In addition, we distinguished several food subgroups known or suspected of concentrating arsenic from the environment: fish (comprising both shellfish and finfish); rice (equivalent to the rice food subgroup defined by the USDA Food Surveys Research Group); rice cakes and crackers; rice beverage and milk; fruit juice and drink; and water (not bottled) consumed at home (i.e., residential tap water). To avoid double counting, the mass consumed in each subgroup was subtracted from the mass consumed in their respective food group. The USDA food codes and logic for apportioning dietary intake are detailed in Table S1.

#### Linear Regression Models

Additional predictors representing potential confounders were included in the regression models: sex, race/ethnicity, serum folate [mg/L], body mass index (BMI), poverty income ratio (PIR; ratio of self-reported family income to the US Census poverty threshold), education, fasting time (time elapsed since participant last ate or drank anything other than water and the time of specimen collection), tobacco/nicotine use in the five days prior to the physical examination, and NHANES study cycle. Information for potential confounders was self-reported, except for BMI (measured during the physical examination), urinary creatinine, and serum folate. Serum selenium and plasma homocysteine were not included because data were available for limited age groups and NHANES study cycles. Educational attainment for 20–84 year-olds was based on self-report, but for 6–19 year-olds educational attainment was based on the highest degree attained among the household reference person and their spouse. The models were stratified by age: children (6–11 years), adolescents (12–19 years), and adults (20–84 years). There were no age predictors in the children and adolescent models, but because the adult models encompassed a relatively broad age range, an age predictor was included with the categories 20–39 years, 40–59 years, and 60–84 years. No predictor for tobacco/nicotine use was included in the 6–11 years models because NHANES does not collect this information for this age group.

Regression models require complete information on all dependent and predictor data for each subject, which were available for about 8,268 of 10,868 participants in NHANES 2003–2010 depending on arsenic species. Missing data may introduce bias to model estimates depending on the degree of non-randomness in the pattern of missingness. To ameliorate this, the original NHANES survey sample weights were adjusted using the WTADJUST subroutine of SUDAAN version 11.0.0, which treats those participants with complete information as a random subsample of the entire sample. The probability of a sampled participant being in this subsample was assumed to be a logistic function of covariates, which was estimated implicitly by WTADJUST in computing the adjusted analysis weights. Because all models reported here used analysis weights adjusted in this manner, results may be considered statistically representative of the NHANES study population [61].

#### Median & 90^th^ Percentile Diets

The sample-weighted percentile mass consumed in each food group adjusted for body weight [kg consumed/kg body weight] was computed by age stratum from NHANES 2003–2010 data to create a nationally representative percentile dietary consumption profile. Then, for each food group, multiplying the percentile mass by the slope for urinary arsenic species estimated from the regression models [nmol/L per kg consumed] produced arsenic species exposure via the percentile diet for the United States [nmol/L per kg body weight]. For this analysis, food group consumption was estimated for the 50^th^ and 90^th^ percentile diets.

### Ethics Statement

The NCHS Research Ethics Review Board (ERB) protected the rights and welfare of NHANES participants. In accordance with Federal regulations, the NCHS ERB reviewed and approved NHANES protocols and any changes made to them: Protocols #98-12, #2005-06, #2011-17, and their respective continuations. Signed informed consent was obtained from each participant or their parent/guardian prior to collecting any data.

## Results

### Summary Statistics and Demographic Differences

There were 10,868 participants ages 6 years and older in NHANES 2003–2010. The proportion of participants with measurements greater than the LOD differed by age stratum and arsenic species: As(III) (2.8–5.3 percent depending on age stratum); As(V) (3.0–4.9 percent); DMA (84.2–87.4 percent); and MMA (29.2–42.4 percent). Since the inorganic species data had such low detection frequency, substantial bias could be introduced to statistical analysis [62], [63]. Therefore, no further results on inorganic species are reported here, although results from statistical analyses analogous to those reported for methylated species are available from the corresponding author upon request.

Table 1 displays the sample-weighted 25^th^, 50^th^, and 75^th^ percentiles for creatinine-adjusted urinary molar concentration [µmol/mol Cre] for each arsenic species by age stratum. Across all age strata, the rank-order for urinary molar concentration was DMA > MMA, and across all arsenic species concentrations, the rank-order for age was children > adults > adolescents. The median molar concentration was highest for DMA in children at 3.52 µmol/mol Cre and lowest for MMA in adolescents at 0.56 µmol/mol Cre. For each methylated arsenic species, the difference in the geometric mean creatinine-adjusted molar concentration was statistically significant between adults *vs*. adolescents and adults *vs.* children.

Tables 2–3 display the sample-weighted 25^th^, 50^th^, and 75th percentiles for creatinine-adjusted molar concentrations of urinary arsenic species by age stratum, race/ethnicity, and sex for the same data used in the regression models reported in Tables 4–5. The statistical significance of differences in urinary arsenic species between levels of each demographic characteristic were evaluated in regression models and controlled for all other variables in the models. For p-values, refer to Tables S2–S3. Urinary DMA was significantly higher in Mexican Americans and Other/Multiracial participants in all age strata compared to non-Hispanic Whites, and significantly elevated among adult and adolescent Other Hispanics. Among adults, males had significantly lower urinary DMA than females. Adults 40–59 years and 60–84 years had significantly elevated urinary DMA compared to 20–39 years participants. Urinary MMA among adults, adolescents, and children was significantly lower in non-Hispanic Blacks compared to non-Hispanic Whites, and significantly elevated among Other Hispanic and Other/Multiracial adults.

**Table 4:** Summary of food groups with DMA regression slope p-values ≤0.15 (results identical to those in Table S2).

		DMA					
Regression Slope		Adults		Adolescents		Children	
Sign	p-Value	Food Group	Slope	Food Group	Slope	Food Group	Slope
**Positive**	**Significant**	Fish	181.16	Rice cakes/crackers	872.55	Rice	115.38
		Rice	105.58	Rice	101.05	Rice cakes/crackers	82.20
		Rice beverage/milk*	84.15	Fish	85.14	Fish	46.99
		Fruits	13.55	Legumes, Nuts, Seeds	24.96	Fruits	33.13
		Legumes, Nuts, Seeds	4.15	Meat, Poultry	7.82	—	—
		Meat, Poultry	2.23	—	—	—	—
		Grain Products	1.88	—	—	—	—
	**Marginally Significant**	—	—	Grain Products	2.37	Meat, Poultry	8.08
**Negative**	**Marginally Significant**	Vegetables	−1.36	Sugars, Sweets, Beverages	−0.72	Sugars, Sweets, Beverages	−3.23
		Milk Products	−2.34	—	—	—	—
	**Significant**	—	—	—	—	—	—

Complete tabulation of model slopes is in Table S2–S3.

Significant: p-Value ≤0.05.

Marginally Significant: 0.05 < p-Value ≤0.15.

*: Data on rice beverage/milk consumption available for adults only during NHANES 2003–2010.

**Table 5:** Summary of food groups with MMA regression slope p-values ≤0.15 (results identical to those in Table S3).

		MMA					
Regression Slope		Adults		Adolescents		Children	
Sign	p-Value	Food Group	Slope	Food Group	Slope	Food Group	Slope
**Positive**	**Significant**	Rice beverage/milk*	6.45	Rice cakes/crackers	103.04	Rice cakes/crackers	65.59
		Rice	5.75	Rice	11.71	Grain Products	2.22
		Fruits	1.24	—	—	—	—
		Sugars, Sweets, Beverages	0.14	—	—	—	—
	**Marginally Significant**	—	—	Milk Products	0.47	Rice	11.10
		—	—	Fruit juice/drink	0.31	Eggs	3.14
**Negative**	**Marginally Significant**	—	—	—	—	Sugars, Sweets, Beverages	−0.40
	**Significant**	Milk Products	−0.69	—	—	—	—

Complete tabulation of model slopes is in Table S2–S3.

Significant: p-Value ≤0.05.

Marginally Significant: 0.05 < p-Value ≤0.15.

*: Data on rice beverage/milk consumption available for adults only during NHANES 2003–2010.

### Food Groups Associated with Urinary Methylated Arsenic

The creatinine-adjusted associations between food group consumed and urinary arsenic species were estimated with sample-weighted regression model slopes, which are displayed in Tables 4–5 (complete tabulation of slopes are in Tables S2–S3), controlling for potential confounders. To facilitate interpretation, Tables 4–5 display only the food groups for slopes with p-values ≤0.15, ordered by magnitude. The creatinine-adjusted food group slopes represent the change in urinary molar arsenic concentration attributable to one additional kilogram consumed of a food group [nmol/L per kg consumed]. The displayed p-values are from identical models except that the dependent variable was ln-transformed. No parameter estimates are available for adolescents and children consuming rice beverage/milk because no NHANES 2003–2010 participants in these age strata reported consuming this food group.

Several continuous potential confounders were influential predictors in the regression models (Tables S2–S3; categorical potential confounders were discussed in the summary statistics section). Urinary creatinine was a positive and statistically significant predictor across all age strata and arsenic species. Serum folate was a significant positive predictor for DMA and MMA among adolescents. BMI was a statistically significant negative predictor for DMA among adults and for MMA among all age strata. Fasting time was a statistically significant negative predictor for DMA among adolescents and a positive predictor for DMA and MMA among adults.

The largest significant increase in urinary molar concentration of methylated arsenic species was for DMA (872.55 nmol/L per kg) attributable to rice cakes/crackers consumed by adolescents (Table 4). The next largest molar increase among methylated species was associated with fish consumption among adults: 181.16 nmol DMA/L per kg. The third largest molar increase was for rice consumption among children at 115.38 nmol DMA/L per kg, followed by 105.58 nmol DMA/L per kg rice consumed by adults. At the fifth rank, MMA appears for the first time with 103.04 nmol MMA/L per kg rice cake/crackers consumed by adolescents (Table 5). The magnitude of association for the remaining food groups associated with significant increases in methylated arsenic ranged from 0.14 nmol MMA/L to 101.05 nmol DMA/L per kg for fish; fruits; grain products; legumes, nuts, seeds; meat, poultry; rice; rice cakes/crackers; rice beverage/milk (adults only); and sugars, sweets, beverages.

Only one food group was associated with a significant decrease in methylated arsenic: −0.69 nmol MMA/L per kg milk products consumed by adults.

### Arsenic Exposure for the Median & 90^th^ Percentile Diets

Arsenic species exposure [nmol/L per kg body weight (bw)] are tabulated for the median (Table 6) and 90^th^ percentile (Table 7) United States diets, where asterisks indicate food groups significantly associated with creatinine-adjusted changes in urinary arsenic species, as shown by the regression model slopes (Tables 4–5). The net total dietary urinary arsenic species for each age stratum is also shown.

**Table 6:** Sample-weighted change in urinary methylated arsenic species [nmol/L per kg body weight] attributable to consuming each food group at the median amount estimated from NHANES 2003–2010 data.

	DMA			MMA		
Food Group	20–84	12–19	6–11	20–84	12–19	6–11
Milk Products	−3.70E-3	−8.21E-3	−7.77E-2	−1.09E-3*	1.37E-3	−4.30E-3
Meat, Poultry	4.17E-3*	1.58E-2*	2.34E-2	−9.46E-4	−4.64E-5	1.16E-3
Eggs	—	—	—	—	—	—
Legumes, Nuts, Seeds	—*	—*	—	—	—	—
Grain Products	5.30E-3*	1.06E-2	7.27E-2	−1.28E-3	2.69E-4	1.73E-2*
Fruits	—*	—	—*	—*	—	—
Vegetables	−2.05E-3	−2.24E-5	−2.20E-2	4.54E-4	−9.62E-4	1.63E-4
Fats, Oils, Salad Dressings	—	—	—	—	—	—
Sugars, Sweets, Beverages	5.07E-3	−8.85E-3	−3.66E-2	2.45E-3*	−2.94E-4	−4.49E-3
Fish	—*	—*	—*	—	—	—
Rice	—*	—*	—*	—*	—*	—
Rice cakes/crackers	—	—*	—*	—	—*	—*
Rice beverage/milk	—*	NA	NA	—*	NA	NA
Fruit juice/drink	—	—	4.84E-3	—	—	2.93E-3
Water (Not Bottled) At Home	—	—	—	—	—	—
**NET TOTAL**	**8.78E-3**	**9.32E-3**	**−3.54E-2**	**−4.14E-4**	**3.35E-4**	**1.28E-2**

*: food group significantly associated with urinary arsenic species in regression models reported in Tables 3a and 3b.

—: food group data insufficient to estimate sample-weighted percentile consumption.

NA: rice beverage/milk was only evaluated in adults 20–84 years because no NHANES participants <20 years reported consuming this food group.

**Table 7:** Sample-weighted change in urinary methylated arsenic species [nmol/L per kg body weight] attributable to consuming each food group at the 90^th^ percentile amount estimated from NHANES 2003–2010 data.

	DMA			MMA		
Food Group	20–84	12–19	6–11	20–84	12–19	6–11
Milk Products	−1.74E-2	−3.45E-2	−2.17E-1	−5.13E-3*	5.75E-3	−1.20E-2
Meat, Poultry	1.32E-2*	5.38E-2*	7.57E-2	−2.99E-3	−1.58E-4	3.74E-3
Eggs	−7.47E-4	2.57E-3	−3.31E-2	−1.69E-3	−3.17E-3	6.61E-3
Legumes, Nuts, Seeds	6.27E-3*	1.95E-2*	2.98E-2	−1.50E-3	1.84E-3	−1.43E-3
Grain Products	1.52E-2*	2.92E-2	1.79E-1	−3.68E-3	7.39E-4	4.26E-2*
Fruits	4.71E-2*	1.31E-3	2.41E-1*	4.29E-3*	−2.67E-4	5.83E-3
Vegetables	−7.39E-3	−1.01E-4	−1.02E-1	1.64E-3	−4.33E-3	7.59E-4
Fats, Oils, Salad Dressings	−1.11E-2	−1.90E-3	3.52E-2	−2.12E-3	−1.81E-3	1.31E-2
Sugars, Sweets, Beverages	1.26E-2	−2.53E-2	−1.00E-1	6.10E-3*	−8.42E-4	−1.23E-2
Fish	7.26E-2*	—*	—*	3.72E-4	—	—
Rice	—*	—*	—*	—*	—*	—
Rice cakes/crackers	—	—*	—*	—	—*	—*
Rice beverage/milk	—*	NA	NA	—*	NA	NA
Fruit juice/drink	−9.83E-4	2.85E-2	1.72E-2	3.13E-3	4.30E-3	1.04E-2
Water (Not Bottled) At Home	−4.59E-3	6.79E-3	−2.29E-2	1.07E-3	−2.10E-3	9.65E-3
**NET TOTAL**	**1.25E-1**	**7.99E-2**	**1.02E-1**	**−5.09E-4**	**−5.92E-5**	**6.69E-2**

*: food group significantly associated with urinary arsenic species in regression models reported in Tables 3a and 3b.

—: food group data insufficient to estimate sample-weighted percentile consumption.

NA: rice beverage/milk was only evaluated in adults 20–84 years because no NHANES participants <20 years reported consuming this food group.

#### Median Diet

The net effect of all food groups on urinary arsenic can be appreciated by combining the regression slopes quantifying the contribution of each food group to urinary arsenic with information on the amount of each food group consumed. For the methylated arsenic species, children (1.28E-2 nmol MMA/L per kg bw), adolescents (9.32E-3 nmol DMA/L and 3.35E-4 nmol MMA/L per kg bw), and adults (8.78E-3 nmol DMA/L per kg bw) had a net increase in urinary arsenic from the median diet (Table 6).

Molar contributions from the median diet to net urinary methylated arsenic species was greatest among children eating grain products (7.27E-2 nmol DMA/L per kg bw, followed by 1.73E-2 nmol MMA/L per kg bw), followed by DMA in adolescents eating meat, poultry (1.58E-2 nmol DMA/L per kg bw). The regression slopes, however, were significant only for children who ate grain products (DMA slope p-value  = 0.0333) and adolescents who ate meat, poultry (DMA slope p-value  = 0.0024).

#### 90^th^ Percentile Diet

Several food groups with positive and statistically significant regression slopes — fish; fruits; legumes, nuts, seeds; rice; rice beverage/milk; rice cakes/crackers; and sugar, sweets, beverages — were not represented or were not estimable in the median dietary consumption profile, most likely due to sparse numbers of NHANES participants who reported consuming these foods. In order to evaluate the influence of high dietary consumption, we examined the 90^th^ percentile dietary consumption profile for the United States (Table 7). Fish; fruits; legumes, nuts, seeds; and sugar, sweets, beverages now appear in the profile, but rice; rice beverage/milk; and rice cakes/crackers were still absent across all arsenic species and age strata.

Reviewing the net changes in urinary arsenic species for the 90^th^ percentile United States diet, methylated arsenic species (Table 7) increased for adults (1.25E-1 nmol DMA/L per kg bw), adolescents (7.99E-2 nmol DMA/L per kg bw), and children (1.02E-1 nmol DMA/L per kg bw and 6.69E-2 nmol MMA/L per kg bw).

Increases in urinary methylated arsenic species for the 90^th^ percentile diet comprised several food groups with positive and statistically significant regression slopes. The greatest molar increase among methylated species was associated with increased fruit consumption among children (2.41E-1 nmol DMA/L per kg bw), followed by fish eaten by adults (7.26E-2 nmol DMA/L per kg bw), meat, poultry eaten by adolescents (5.38E-2 nmol DMA/L per kg bw), fruits eaten by adults (4.71E-2 nmol DMA/L per kg bw), grain products eaten by children (4.26E-2 nmol MMA/L per kg bw), and legumes, nuts, seeds eaten by adolescents (1.95E-2 nmol DMA/L per kg bw). The next largest increase was associated with grain products consumed by adults (1.52E-2 nmol DMA/L per kg bw), followed by meat, poultry (1.32E-2 nmol DMA/L per kg bw), legumes, nuts, seeds (6.27E-3 nmol DMA/L per kg bw), sugars, sweets, beverages (6.10E-3 nmol MMA/L per kg bw), and fruits (4.29E-3 nmol MMA/L per kg bw).

## Discussion

This analysis provides the most comprehensive model of NHANES data characterizing the association of food consumption with methylated urinary arsenic, while accounting for numerous potential confounders (urinary creatinine, serum folate, sex, race, BMI, poverty income ratio, education, fasting time, recent tobacco/nicotine use, and NHANES study cycle). Our analysis details which food groups contribute to each urinary arsenic species and how these dietary contributions vary among age groups in the US population. Similar to previous studies examining populations without high arsenic exposure from drinking water, we found that increased rice and fish consumption were associated with increased urinary methylated arsenic [36], [41]–[44], [64]. We also found that consuming either home tap water or fruit juice/drink was not significantly associated with any of the two urinary arsenic species (Tables S2–S3). The absence of a significant association with home tap water consumption reflects the comparatively low concentration of arsenic in United States drinking water, which averages 2 µg/L [9]. In addition, we assessed urinary arsenic excretion attributable to dietary sources based on 50th and 90th percentile rates of food consumption for the US population in combination with the rates of dietary arsenic exposure estimated in the regression models.

The methylated arsenic species selected for analysis are associated with public health concerns for potential carcinogenicity and other adverse health effects [1], [2], [65]. Of the two methylated species, DMA was observed at the highest concentrations and detected among the most participants. Rice, rice cakes/crackers, and fish were the most prominent sources of urinary DMA (Table 4). Other food groups that contributed significantly to urinary DMA, but to a lesser degree, were fruits (adults, children); meat, poultry (adults, adolescents); and legumes, nuts, seeds (adults, adolescents). For urinary MMA, the most prominent contributions came from rice cakes/crackers eaten by adolescents and children, followed by adults consuming rice beverage/milk and rice (Table 5). Other food groups that contributed significantly to urinary MMA were fruits (adults) and sugars, sweets, beverages (adults).

The food groups and the magnitudes of their contributions to urinary arsenic species vary markedly with age. For example, increased fish consumption was associated with the greatest change in urinary DMA among adults (181.16 nmol DMA/L per kg), but diminished to 85.14 nmol DMA/L per kg among adolescents, then to 46.99 nmol DMA/L per kg among children. The greatest increase in urinary DMA associated with increased consumption of rice cakes/crackers was among adolescents (872.55 nmol DMA/L per kg), but diminished sharply to 82.20 nmol DMA/L per kg among children.

Age-dependent variations like these may be due to variation in the types and amounts of food consumed, and although the USDA food classification scheme standardizes references to the diverse cornucopia of food consumed throughout the United States, the composition of food in each category, which in some cases is broad, could vary considerably in arsenic content. Consider, for example, that arsenic in soil varies spatially in relation to underlying geology, or through anthropogenic deposition associated with such activities as pesticide application, mining, discharge of metal processing waste, and fossil fuel combustion [66], [67]. The same type of food harvested in one area may therefore have a different arsenic species concentration profile than another area. Rice, for example, varies markedly in arsenic species content depending on where it is obtained [68], or whether it is brown or white (polished) rice [69], [70]. In the south central United States, arsenic is present in soil where arsenical pesticides were applied to cotton crops for decades since the 1930s [71]–[73]. This acreage has been given over to rice varieties that provide favorable yields in arsenical-pesticide-treated soils [74], [75], and the relatively high arsenic content of rice grown in this region may be attributable to historical arsenical pesticide use [40], [76]. Such compositional variations in dietary exposure may vary sufficiently with age to affect the statistical association of aggregate food groups with urinary arsenic species.

Indeed, the potential for exploring exposure to arsenic species from the numerous USDA food groups represented in NHANES data has not been exhausted by this analysis. Yet, while examining narrower food group designations would yield greater specificity, this would be counterbalanced by diminishing mass attributable to the food group.

The geographic and age-dependent variation just discussed also introduces a degree of exposure misclassification that reduces the overall statistical power of the study, and this reduction may be accentuated for food groups that comprise a relatively small proportion of the diet or a small population subgroup. Similarly, recall data is less precise than objectively measuring, say, the amount of arsenic species consumed via each food group [77].

The proportion of analyzed data with urinary concentrations exceeding the LOD ranged from 29.2 percent for MMA in children to 87.4 percent for DMA in adolescents (Tables 2–3). Diminished detection frequency potentially admits statistical bias, especially since measurements below the LOD were substituted with a fixed value (the quotient of the LOD divided by the square-root of 2) [62], [63]. In a similar vein, the data are limited to the extent that NHANES participants consumed sufficient quantities of each food group to detect a significant change in urinary arsenic species. Since food groups are consumed disproportionately, it is possible that underrepresented food groups that were found in the regression models to not have significant influence on urinary arsenic species may nevertheless be important dietary sources of arsenic exposure. The clearest example of this was for rice beverage/milk, which was not reported consumed by adolescents and children in the NHANES 2003–2010 cycles and so its contribution to arsenic exposure could not be evaluated.

Another consideration is that food groups found to have significant influence on urinary arsenic species may not be consumed in quantities sufficient to appreciably affect population exposure. Conversely, food groups with relatively low rates of association with urinary arsenic may be consumed in great enough quantities to affect population exposure. We also found that increased consumption of some food groups was associated with reductions in urinary arsenic species, so the net effect on urinary arsenic would be expected to depend on the mix of food groups in a population's diet. Alternatively, it is possible the reductions apparent in the regression model results could have arisen from bias associated with substituting measurements below LOD with the quotient of the LOD divided by the square-root of two.

To evaluate the influence of population food consumption patterns on arsenic exposure, we combined urinary arsenic regression slopes for food consumption with statistical estimates of US population consumption of each food group. This approach highlighted a different set of food groups. For people who eat food groups at typical rates, as represented by the median diet (Table 6), increases in urinary arsenic species among adults were significantly attributable to meat, poultry (DMA); grain products (DMA); and sugars, sweets, beverages (MMA). Among adolescents, increases in urinary arsenic species were significantly attributable to meat, poultry (DMA). Among children, increases in urinary arsenic species were significantly attributable to grain products (MMA). For people who eat certain food groups at high rates, as represented by the 90^th^ percentile diet (Table 7), increases in urinary arsenic species among adults were significantly attributable to fish (DMA); fruits (DMA, MMA); grain products (DMA); legumes, nuts, seeds (DMA); meat, poultry (DMA); and sugars, sweets, beverages (MMA). Among adolescents, increases in urinary arsenic species were significantly attributable to legumes, nuts, seeds (DMA) and meat, poultry (DMA), and among children to fruits (DMA) and grain products (MMA).

**Figure 1:**
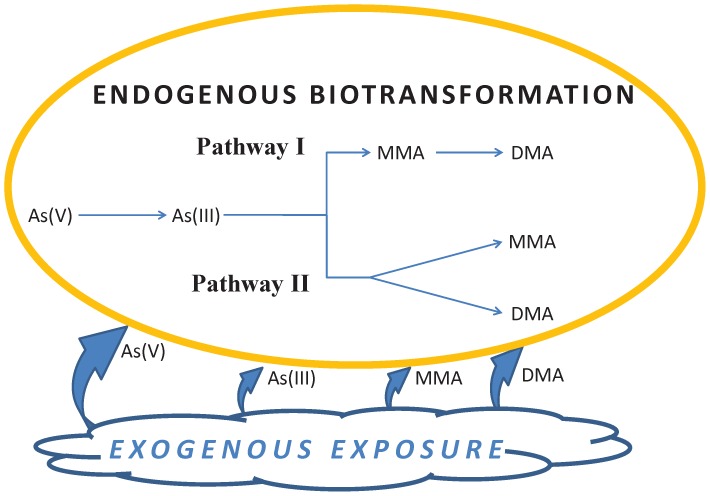
Biotransformation pathways for arsenic species As(III), As(V), DMA, and MMA.

Figure 1 displays inorganic and methylated arsenic species on a diagram depicting two biotransformation pathways adapted from Figures 3–7 and 3–8 of ATSDR's Toxicological Profile for Arsenic [9], which in turn were adapted from Aposhian et al. [78] (Pathway I), as well as Hayakawa et al. [79] and Thomas et al. [80] (Pathway II). Although determination of the exact metabolic pathway(s) for the methylation of inorganic arsenic is still the subject of study [81], simple attribution of speciated arsenic exposure to particular food groups is clouded by the metabolic interrelationship depicted in Figure 1. The concentration of each species may increase either directly by exposure to exogenous dietary or non-dietary sources, or — for As(III), DMA, and MMA — indirectly by endogenous biotransformation from precursors. Alternatively, arsenic species may decrease by endogenous biotransformation to subsequent metabolites. It is therefore impossible to identify with NHANES data whether the source of measured arsenic species is exogenous or endogenous. For example, since methylated arsenic species are end products of the biotransformation pathways, it would have been desirable if their concentration — expressed perhaps as DMA or DMA + MMA — could be interpreted as a measure of cumulative exposure to inorganic arsenic species, which have usually been of greatest human health concern. Yet, because methylated species may arise either from endogenous biotransformation of inorganic species or from environmental exposure, it cannot be said that methylated species concentration is a simple proportional measure of risk from inorganic species. Conversely, the low detection frequency of inorganic arsenic species among NHANES 2003–2010 participants does not necessarily indicate an absence of exposure to these species from diet. While this difficulty in discriminating endogenous from exogenous sources may admittedly be irrelevant to the ultimate risk posed by all arsenic exposure, it nevertheless complicates attribution of arsenic species to specific dietary sources.

Rice is prominent in certain ethnic cuisines, most notably in Spanish and Asian cooking, while fish is a staple of Asian cooking. Racial/ethnic patterns in molar concentrations of urinary arsenic species were compared to non-Hispanic Whites in the linear regression models, controlling for all other predictors in the models (Tables S2–S3). Mexican Americans across all age strata had significantly elevated urinary DMA molar concentrations compared to non-Hispanic Whites. Other Hispanic adults and adolescents had significantly elevated urinary DMA molar concentrations, while Other Hispanic adults had significantly elevated urinary MMA. Also, although comprising a relatively small number of participants, Other/Multi-Racial participants (which include those of Asian ethnicity) across all age strata had significantly elevated urinary DMA, while Other/Multi-Racial adults had significantly elevated urinary MMA molar concentrations. These racial/ethnic elevations may be attributable to ethnic dietary patterns favoring arsenic-rich food groups like rice and fish (although diet would have been accounted for to a fair degree by the food group predictors included in the regression models), racial/ethnic differences in biotransformation of arsenic species, or a combination. Further research is needed to elucidate the causes for these racial/ethnic differences.

We evaluated the relative impact of different demographic and dietary factors on urinary arsenic species. Including urinary creatinine concentration as a predictor variable in all regression models accounted for varying rates of fluid consumption and excretion, as well as varying duration of when the urine specimen accumulated before micturition [62], [82]. Additionally, fasting time and serum folate were included in the regression models and found to be statistically significant predictors in several models. Serum folate's statistical significance was consistent with its demonstrated role in metabolic methylation of arsenic [21]. Other contemporary analyses of NHANES data examining dietary exposure to arsenic have omitted fasting time and serum folate [42], [43], [83], [84]. In addition, although availability of NHANES data on selenium and serum homocysteine was too restrictive for the scope of our analysis, future research should consider including these measurements because of their role in arsenic metabolism [17], [19], [21], [22].

The large size of the NHANES sample used for this study — approximately 8,300 participants— not only permitted control for several potential confounders in the statistical analysis, but also allowed age-stratified analyses, which revealed different excretion patterns among age strata. The data used in this report also comprise the largest number of NHANES cycles used for examining dietary exposure to arsenic compared to other contemporary analyses [42], [43], [83], [84].

## Conclusions

In a comprehensive evaluation of dietary sources of arsenic exposure in a representative sample of the United States civilian, non-institutionalized population observed 2003–2010, certain food groups were found to have been significantly associated with increases in urinary methylated arsenic species concentration: fish (DMA); fruits (DMA, MMA); grain products (DMA, MMA); legumes, nuts, seeds (DMA); meat, poultry (DMA); rice (DMA, MMA); rice cakes/crackers (As(III), DMA, MMA); sugars, sweets, beverages (MMA). Rice beverage/milk (DMA, MMA) contributed significantly among adults, but this food group could not be evaluated for the other age strata because child and adolescent NHANES participants during the 2003–2010 study cycles did not report consuming this food group. Different food groups in each age stratum contributed to each arsenic species with differing magnitudes. Fruit juice/drinks and home tap water were not significantly associated with changes in urinary arsenic.

This study also examined how dietary exposure to arsenic species depends on the amount consumed of each food group, as represented by typical (median) and high (90^th^ percentile) dietary intake patterns in the United States. This perspective focused attention on a subset of food groups. At typical (median) dietary intakes, statistically significant sources of arsenic species were: grain products; meat, poultry; and sugars, sweets, beverages. At high (90^th^ percentile) dietary intakes, statistically significant sources of arsenic species were: fish; fruits; grain products; legumes, nuts, seeds; meat, poultry; and sugars, sweets, beverages. Dietary intakes of these food groups were also found to vary between age strata.

## Supporting Information

Table S1 **USDA Food Codes and Logic for Apportioning Dietary Intake.** To prepare the data for analysis, we summed the mass consumed in each food group so that each participant was represented by a single record describing their dietary intake for the previous 24 hours. Each participant's dietary intake was first apportioned over nine food groups: milk products; meat, poultry; eggs; legumes, nuts, seeds; grain products; fruits; vegetables; fats, oils, salad dressings; and sugars, sweets, beverages. In addition, we distinguished several food subgroups known or suspected of concentrating arsenic from the environment: fish (USDA food codes 261, 262, and 263, comprising both shellfish and finfish); rice (comprising multiple food codes equivalent to the rice food subgroup defined by the USDA Food Surveys Research Group); rice cakes and crackers (comprising multiple food codes); rice beverage and milk (USDA food code 92205000); and fruit juice and drink (comprising multiple food codes). Each of these subgroups is a constituent of a broader food group in the USDA food code hierarchy, so to avoid double counting the mass consumed in these subgroups was subtracted from the mass consumed in their respective food group. The food codes and logic for apportioning dietary intake are detailed in Table S1. We distinguished one more food subgroup of special interest as a route of arsenic exposure: water (not bottled) consumed at home (i.e., residential tap water) as an additional food subgroup. This subgroup was identified when the food code equaled 940 and the subject answered “yes” to whether water (not bottled) was consumed at home. Water (not bottled) is a constituent of the sugars, sweets, and beverages group (USDA food code 9), so for each participant, the mass consumed at home of water (not bottled) was subtracted from the mass consumed of the sugars, sweets, and beverages group to avoid double counting.
(DOCX)

Table S2 **Change in urinary DMA [nmol/L per kg] attributable to mass consumed estimated from sample-weighted, multiple regression models of NHANES 2003–2010 data.** Estimates adjusted for urine volume by including urinary creatinine as a predictor in the models. Slopes are in units of nmol arsenic species/L per kg food consumed. CI: confidence interval HS: high school. NA: rice beverage/milk was only evaluated in adults 20–84 years old because no NHANES participants <20 years old reported consuming this food group. YO: years-old. a: p-Values estimated from identical models where the dependent variable was ln-transformed urinary arsenic species. Hypothesis tests represent comparisons with a slope equal to zero.
(DOCX)

Table S3 **Change in urinary MMA [nmol/L per kg] attributable to mass consumed estimated from sample-weighted, multiple regression models of NHANES 2003–2010 data.** Estimates adjusted for urine volume by including urinary creatinine as a predictor in the models. Slopes are in units of nmol arsenic species/L per kg food consumed. CI: confidence interval. HS: high school. NA: rice beverage/milk was only evaluated in adults 20–84 years old because no NHANES participants <20 years old reported consuming this food group. YO: years-old. a: p-Values estimated from identical models where the dependent variable was ln-transformed urinary arsenic species. Hypothesis tests represent comparisons with a slope equal to zero.
(DOCX)
