# Supplementary material for: Dietary Sources of Methylated Arsenic Species in Urine of the United States Population, NHANES 2003–2010
Source: PLoS One. 2014 Sep 24;9(9):e108098. doi: 10.1371/journal.pone.0108098 (PMC4176478; doi:10.1371/journal.pone.0108098)
Supplement: Table S1 — USDA Food Codes and Logic for Apportioning Dietary Intake. To prepare the data for analysis, we summed the mass consumed in each food group so that each participant was represented by a single record describing their dietary intake for the previous 24 hours. Each participant's dietary intake was first apportioned over nine food groups: milk products; meat, poultry; eggs; legumes, nuts, seeds; grain products; fruits; vegetables; fats, oils, salad dressings; and sugars, sweets, beverages. In addition, we distinguished several food subgroups known or suspected of concentrating arsenic from the environment: fish (USDA food codes 261, 262, and 263, comprising both shellfish and finfish); rice (comprising multiple food codes equivalent to the rice food subgroup defined by the USDA Food Surveys Research Group); rice cakes and crackers (comprising multiple food codes); rice beverage and milk (USDA food code 92205000); and fruit juice and drink (comprising multiple food codes). Each of these subgroups is a constituent of a broader food group in the USDA food code hierarchy, so to avoid double counting the mass consumed in these subgroups was subtracted from the mass consumed in their respective food group. The food codes and logic for apportioning dietary intake are detailed in Table S1. We distinguished one more food subgroup of special interest as a route of arsenic exposure: water (not bottled) consumed at home (i.e., residential tap water) as an additional food subgroup. This subgroup was identified when the food code equaled 940 and the subject answered “yes” to whether water (not bottled) was consumed at home. Water (not bottled) is a constituent of the sugars, sweets, and beverages group (USDA food code 9), so for each participant, the mass consumed at home of water (not bottled) was subtracted from the mass consumed of the sugars, sweets, and beverages group to avoid double counting. (DOCX) [file pone.0108098.s001.docx]

# SUPPLEMENTAL TABLE S1

| Food Group | USDA Food Code and Additional Logic |
| --- | --- |
| Milk and Milk Products | DR1IFDCD = 1 |
| Meat, Poultry, and Mixtures | DR1IFDCD = 2 |
| Eggs | DR1IFDCD = 3 |
| Legumes, Nuts, and Seeds | DR1IFDCD = 4 |
| Grain Products | DR1IFDCD = 5 |
| Fruits | DR1IFDCD = 6 |
| Vegetables | DR1IFDCD = 7 |
| Fats, Oils, and Salad Dressings | DR1IFDCD = 8 |
| Sugars, Sweets, and Beverages | DR1IFDCD = 9 |
|  |  |
| Fish | DR1IFDCD = 261, 262, or 263  Then subtract total mass from DR1IFDCD = 2 |
|  |  |
| Rice  Equal to FSRG-defined food group: GRAIN22 (rice) | DR1IFDCD = 562049, 562050, 562051, 562053, 562054, 562055, 5620521, or 57603  Then subtract total mass from DR1IFDCD = 5 |
|  |  |
| Rice cakes/crackers  Includes puffed rice cake, rice crackers, and popcorn cake | DR1IFDCD = 54206010, 54318500, 54319000, 54319010, or 54319020  Then subtract total mass from DR1IFDCD = 5 |
|  |  |
| Fruit juice/drink  Combines USDA FSRG-defined FRUIT11 (citrus juices), FRUIT35 (noncitrus juices and nectars), BEV23 (total fruit juice drinks and fruit flavored drinks), as well as fruit juice not further specified. | DR1IFDCD = 61201 through 61213, 6410011 through 6422101, 67202 through 67204, 67211 through 67260, or 6720500  Then subtract total mass from DR1IFDCD = 6  *OR*  DR1IFDCD = 925 or 929  Then subtract total mass from DR1IFDCD = 9 |
| Rice beverage/milk  Rice beverage: Rice tea, rice Dream beverage, rice "milk" | DR1IFDCD = 92205000  Then subtract total mass from DR1IFDCD = 9 |
|  |  |
| Water, Not Bottled consumed at home  Water as an ingredient  Water, tap | DR1IFDCD = 940  *AND*  DR1_040Z = 1 (Answered “Yes” to “Did you eat this meal at home?”)  Then subtract total mass from DR1IFDCD = 9 |
