# Supplementary material for: Dietary Sources of Methylated Arsenic Species in Urine of the United States Population, NHANES 2003–2010
Source: PLoS One. 2014 Sep 24;9(9):e108098. doi: 10.1371/journal.pone.0108098 (PMC4176478; doi:10.1371/journal.pone.0108098)
Supplement: Table S2 — Change in urinary DMA [nmol/L per kg] attributable to mass consumed estimated from sample-weighted, multiple regression models of NHANES 2003–2010 data. Estimates adjusted for urine volume by including urinary creatinine as a predictor in the models. Slopes are in units of nmol arsenic species/L per kg food consumed. CI: confidence interval HS: high school. NA: rice beverage/milk was only evaluated in adults 20–84 years old because no NHANES participants <20 years old reported consuming this food group. YO: years-old. a: p-Values estimated from identical models where the dependent variable was ln-transformed urinary arsenic species. Hypothesis tests represent comparisons with a slope equal to zero. (DOCX) [file pone.0108098.s002.docx]

# SUPPLEMENTAL TABLE S2

|  | **Adults (20 – 84 YO)** | | **Adolescents (12 – 19 YO)** | | **Children (6 – 11 YO)** | |
| --- | --- | --- | --- | --- | --- | --- |
| **Predictor** | **Slope (95% CI)** | **p-Value^a^** | **Slope (95% CI)** | **p-Value^a^** | **Slope (95% CI)** | **p-Value^a^** |
| Intercept | 12.65 (2.37, 22.93) | <.0001 | 8.00 (-1.87, 17.87) | <.0001 | 25.71 (4.32, 47.11) | <.0001 |
| Creatinine, urine [g/L] | 24.12 (21.19, 27.05) | <.0001 | 16.21 (14.22, 18.21) | <.0001 | 25.43 (20.07, 30.80) | <.0001 |
| Folate, serum [mg/L] | -51.23 (-122.87, 20.41) | 0.8053 | 232.56 (20.70, 444.42) | 0.0278 | -54.55 (-492.83, 383.74) | 0.4233 |
| Sex |  |  |  |  |  |  |
| Male | -5.48 (-8.72, -2.25) | <.0001 | -1.25 (-4.12, 1.61) | 0.6523 | -3.61 (-12.39, 5.17) | 0.5770 |
| Female | Ref. |  | Ref. |  | Ref. |  |
| Age at Screening |  |  |  |  |  |  |
| 20 - 39 YO | Ref. |  |  |  |  |  |
| 40 - 59 YO | 5.30 (1.78, 8.82) | <.0001 |  |  |  |  |
| 60 - 84 YO | 7.60 (4.35, 10.85) | <.0001 |  |  |  |  |
| Race/Ethnicity |  |  |  |  |  |  |
| Mexican American | 10.34 (4.75, 15.93) | <.0001 | 6.31 (2.61, 10.01) | <.0001 | 5.64 (0.19, 11.09) | 0.0007 |
| Other Hispanic | 22.60 (13.67, 31.53) | <.0001 | 11.66 (4.29, 19.03) | 0.0193 | 1.03 (-6.86, 8.91) | 0.2006 |
| Non-Hispanic White | Ref. |  | Ref. |  | Ref. |  |
| Non-Hispanic Black | 0.42 (-4.19, 5.03) | 0.1934 | -0.15 (-5.60, 5.30) | 0.3142 | -0.68 (-9.56, 8.20) | 0.5340 |
| Other/Multi-Racial | 20.23 (11.96, 28.51) | <.0001 | 14.25 (1.79, 26.70) | 0.0044 | 23.42 (1.54, 45.31) | 0.0078 |
| Education |  |  |  |  |  |  |
| HS Graduate or Higher | Ref. |  | Ref. |  | Ref. |  |
| Less Than HS Graduate | -0.84 (-4.50, 2.82) | 0.6148 | -3.23 (-6.47, 0.02) | 0.0991 | -6.48 (-11.24, -1.72) | 0.1240 |
| Poverty Income Ratio |  |  |  |  |  |  |
| 1.00 or Higher | Ref. |  | Ref. |  | Ref. |  |
| <1.00 (Poor) | 0.29 (-4.53, 5.11) | 0.2210 | -1.05 (-4.25, 2.15) | 0.3534 | -1.29 (-9.46, 6.88) | 0.2894 |
| Body Mass Index | -0.39 (-0.64, -0.15) | 0.0005 | -0.07 (-0.30, 0.16) | 0.5303 | -0.72 (-1.51, 0.07) | 0.1020 |
| Fasting Time | 0.22 (-0.01, 0.45) | 0.0441 | -0.25 (-0.45, -0.05) | 0.0093 | -0.51 (-1.01, -0.01) | 0.0613 |
| Tobacco/Nicotine Last 5 Days |  |  |  |  |  |  |
| No | Ref. |  | Ref. |  |  |  |
| Yes | -0.28 (-4.46, 3.91) | 0.0699 | -0.59 (-5.01, 3.83) | 0.6791 |  |  |
| NHANES Cycle |  |  |  |  |  |  |
| 2003-2004 | -3.63 (-10.17, 2.91) | 0.3645 | -1.57 (-8.38, 5.23) | 0.2571 | -4.85 (-10.61, 0.90) | 0.0570 |
| 2005-2006 | Ref. |  | Ref. |  | Ref. |  |
| 2007-2008 | -5.34 (-10.48, -0.21) | 0.1365 | -7.75 (-12.92, -2.58) | 0.0025 | 7.71 (-8.02, 23.44) | 0.9615 |
| 2009-2010 | -2.23 (-7.94, 3.48) | 0.0746 | -6.08 (-11.35, -0.82) | 0.0013 | 6.37 (-4.71, 17.44) | 0.3445 |
| Food Group Mass Consumed [kg] |  |  |  |  |  |  |
| Milk Products | -2.34 (-6.18, 1.49) | 0.0503 | -2.85 (-6.02, 0.33) | 0.1546 | -8.25 (-18.47, 1.97) | 0.1625 |
| Meat, Poultry | 2.23 (-3.54, 8.00) | 0.0030 | 7.82 (-0.51, 16.16) | 0.0024 | 8.08 (-12.19, 28.34) | 0.1465 |
| Eggs | -0.58 (-28.20, 27.03) | 0.6979 | 2.37 (-20.25, 25.00) | 0.4659 | -15.74 (-60.67, 29.19) | 0.7416 |
| Legumes, Nuts, Seeds | 4.15 (-8.68, 16.97) | 0.0110 | 24.96 (2.82, 47.10) | 0.0038 | 28.57 (-36.01, 93.16) | 0.2192 |
| Grain Products | 1.88 (-3.36, 7.12) | 0.0094 | 2.37 (-1.82, 6.55) | 0.0623 | 9.33 (-5.94, 24.60) | 0.1651 |
| Fruits | 13.55 (5.24, 21.86) | <.0001 | 0.38 (-5.45, 6.20) | 0.4543 | 33.13 (-27.29, 93.54) | 0.0172 |
| Vegetables | -1.36 (-8.44, 5.72) | 0.0871 | -0.02 (-11.06, 11.02) | 0.3608 | -13.95 (-36.39, 8.49) | 0.5943 |
| Fats, Oils, Salad Dressings | -21.39 (-86.46, 43.68) | 0.4495 | -6.05 (-70.08, 57.98) | 0.8450 | 68.60 (-379.78, 516.99) | 0.6811 |
| Sugars, Sweets, Beverages | 0.30 (-0.90, 1.49) | 0.5879 | -0.72 (-2.75, 1.31) | 0.0644 | -3.23 (-8.38, 1.92) | 0.0663 |
| Fish | 181.16 (136.57, 225.75) | <.0001 | 85.14 (53.14, 117.13) | <.0001 | 46.99 (-52.06, 146.05) | 0.0075 |
| Rice | 105.58 (71.10, 140.06) | <.0001 | 101.05 (52.60, 149.49) | <.0001 | 115.38 (47.00, 183.76) | <.0001 |
| Rice cakes/crackers | -54.18 (-242.43, 134.06) | 0.2161 | 872.55 (120.82, 1624.28) | 0.0009 | 82.20 (-277.14, 441.53) | 0.0112 |
| Rice beverage/milk | 84.15 (-29.98, 198.28) | 0.0254 | NA |  | NA |  |
| Fruit juice/drink | -0.13 (-3.29, 3.02) | 0.2627 | 2.04 (-0.89, 4.97) | 0.2091 | 0.83 (-8.00, 9.65) | 0.8147 |
| Water (Not Bottled) At Home | -0.28 (-1.61, 1.05) | 0.6874 | 0.52 (-1.51, 2.54) | 0.9561 | -1.42 (-10.77, 7.93) | 0.7833 |
