# Supplementary material for: Dietary Sources of Methylated Arsenic Species in Urine of the United States Population, NHANES 2003–2010
Source: PLoS One. 2014 Sep 24;9(9):e108098. doi: 10.1371/journal.pone.0108098 (PMC4176478; doi:10.1371/journal.pone.0108098)
Supplement: Table S3 — Change in urinary MMA [nmol/L per kg] attributable to mass consumed estimated from sample-weighted, multiple regression models of NHANES 2003–2010 data. Estimates adjusted for urine volume by including urinary creatinine as a predictor in the models. Slopes are in units of nmol arsenic species/L per kg food consumed. CI: confidence interval. HS: high school. NA: rice beverage/milk was only evaluated in adults 20–84 years old because no NHANES participants <20 years old reported consuming this food group. YO: years-old. a: p-Values estimated from identical models where the dependent variable was ln-transformed urinary arsenic species. Hypothesis tests represent comparisons with a slope equal to zero. (DOCX) [file pone.0108098.s003.docx]

# SUPPLEMENTAL TABLE S3

|  | **Adults** | | **Adolescents** | | **Children** | |
| --- | --- | --- | --- | --- | --- | --- |
| **Predictor** | **Slope (95% CI)** | **p-Value^a^** | **Slope (95% CI)** | **p-Value^a^** | **Slope (95% CI)** | **p-Value^a^** |
| Intercept | 6.27 (4.30, 8.24) | <.0001 | 4.74 (2.93, 6.54) | <.0001 | 5.27 (2.87, 7.67) | <.0001 |
| Creatinine, urine [g/L] | 2.60 (2.21, 2.99) | <.0001 | 2.63 (2.03, 3.23) | <.0001 | 2.46 (1.79, 3.13) | <.0001 |
| Folate, serum [mg/L] | -12.13 (-34.09, 9.83) | 0.5777 | 43.77 (0.85, 86.68) | 0.0407 | 29.05 (-26.38, 84.49) | 0.2985 |
| Sex |  |  |  |  |  |  |
| Male | -0.01 (-0.59, 0.57) | 0.8939 | 0.34 (-0.26, 0.94) | 0.0918 | -0.07 (-0.82, 0.68) | 0.3920 |
| Female | Ref. |  | Ref. |  | Ref. |  |
| Age at Screening |  |  |  |  |  |  |
| 20 - 39 YO | Ref. |  |  |  |  |  |
| 40 - 59 YO | 0.57 (-0.05, 1.18) | 0.3776 |  |  |  |  |
| 60 - 84 YO | 0.73 (-0.05, 1.51) | 0.1303 |  |  |  |  |
| Race/Ethnicity |  |  |  |  |  |  |
| Mexican American | 0.89 (-0.36, 2.13) | 0.1543 | 0.51 (-0.37, 1.40) | 0.2496 | 0.46 (-0.31, 1.22) | 0.2428 |
| Other Hispanic | 0.87 (0.10, 1.64) | 0.0038 | 1.75 (-0.84, 4.34) | 0.2269 | -0.00 (-1.39, 1.39) | 0.7375 |
| Non-Hispanic White | Ref. |  | Ref. |  | Ref. |  |
| Non-Hispanic Black | -0.69 (-2.08, 0.69) | <.0001 | -0.79 (-1.49, -0.10) | 0.0125 | -0.88 (-1.79, 0.04) | 0.0358 |
| Other/Multi-Racial | 0.82 (0.11, 1.53) | 0.0047 | 1.74 (0.31, 3.18) | 0.0608 | -0.39 (-1.25, 0.47) | 0.8449 |
| Education |  |  |  |  |  |  |
| HS Graduate or Higher | Ref. |  | Ref. |  | Ref. |  |
| Less Than HS Graduate | 0.18 (-0.53, 0.89) | 0.8163 | -0.56 (-1.24, 0.12) | 0.0381 | -0.52 (-1.19, 0.15) | 0.1146 |
| Poverty Income Ratio |  |  |  |  |  |  |
| 1.00 or Higher | Ref. |  | Ref. |  | Ref. |  |
| <1.00 (Poor) | 0.16 (-0.59, 0.90) | 0.7660 | -0.36 (-0.99, 0.27) | 0.4752 | 0.15 (-0.68, 0.98) | 0.6135 |
| Body Mass Index | -0.10 (-0.15, -0.05) | <.0001 | -0.07 (-0.11, -0.03) | 0.0004 | -0.12 (-0.19, -0.06) | <.0001 |
| Fasting Time | 0.04 (0.00, 0.08) | 0.0041 | 0.01 (-0.04, 0.07) | 0.0875 | 0.02 (-0.06, 0.09) | 0.7212 |
| Tobacco/Nicotine Last 5 Days |  |  |  |  |  |  |
| No | Ref. |  | Ref. |  |  |  |
| Yes | 0.25 (-0.57, 1.08) | 0.7109 | 0.18 (-0.90, 1.25) | 0.7256 |  |  |
| NHANES Cycle |  |  |  |  |  |  |
| 2003-2004 | -0.59 (-1.81, 0.64) | 0.1904 | -0.21 (-2.15, 1.72) | 0.1726 | -0.52 (-1.46, 0.43) | 0.1236 |
| 2005-2006 | Ref. |  | Ref. |  | Ref. |  |
| 2007-2008 | -0.47 (-1.47, 0.54) | 0.3154 | -1.04 (-1.97, -0.11) | 0.0347 | 0.14 (-1.20, 1.48) | 0.9517 |
| 2009-2010 | -0.27 (-1.41, 0.87) | 0.0793 | -1.38 (-2.37, -0.40) | 0.0033 | -0.94 (-2.13, 0.25) | 0.1620 |
| Food Group Mass Consumed [kg] |  |  |  |  |  |  |
| Milk Products | -0.69 (-1.11, -0.27) | 0.0015 | 0.47 (-0.26, 1.21) | 0.1439 | -0.46 (-1.71, 0.80) | 0.6082 |
| Meat, Poultry | -0.51 (-1.19, 0.18) | 0.6115 | -0.02 (-1.55, 1.50) | 0.7372 | 0.40 (-1.88, 2.68) | 0.8196 |
| Eggs | -1.32 (-4.99, 2.35) | 0.1737 | -2.93 (-7.15, 1.28) | 0.1614 | 3.14 (-2.12, 8.41) | 0.0673 |
| Legumes, Nuts, Seeds | -0.99 (-2.60, 0.62) | 0.6117 | 2.35 (-1.63, 6.34) | 0.2588 | -1.37 (-5.32, 2.58) | 0.5458 |
| Grain Products | -0.45 (-1.18, 0.27) | 0.6935 | 0.06 (-1.07, 1.19) | 0.6782 | 2.22 (0.38, 4.06) | 0.0333 |
| Fruits | 1.24 (0.15, 2.32) | 0.0015 | -0.08 (-1.06, 0.91) | 0.8003 | 0.80 (-2.08, 3.68) | 0.3452 |
| Vegetables | 0.30 (-0.64, 1.25) | 0.2235 | -0.95 (-2.86, 0.96) | 0.6048 | 0.10 (-1.91, 2.12) | 0.9217 |
| Fats, Oils, Salad Dressings | -4.08 (-9.99, 1.83) | 0.6325 | -5.77 (-15.83, 4.29) | 0.3051 | 25.47 (-10.80, 61.74) | 0.1545 |
| Sugars, Sweets, Beverages | 0.14 (-0.03, 0.31) | 0.0125 | -0.02 (-0.35, 0.31) | 0.6348 | -0.40 (-1.05, 0.25) | 0.1288 |
| Fish | 0.93 (-2.17, 4.03) | 0.1704 | -2.07 (-5.11, 0.96) | 0.1502 | -4.38 (-11.11, 2.35) | 0.4023 |
| Rice | 5.75 (2.92, 8.58) | <.0001 | 11.71 (6.87, 16.54) | <.0001 | 11.10 (-2.78, 24.99) | 0.0693 |
| Rice cakes/crackers | -21.03 (-44.21, 2.16) | 0.5420 | 103.04 (-4.09, 210.16) | <.0001 | 65.59 (44.18, 87.00) | <.0001 |
| Rice beverage/milk | 6.45 (0.88, 12.01) | 0.0145 | NA |  | NA |  |
| Fruit juice/drink | 0.42 (-0.21, 1.06) | 0.1943 | 0.31 (-0.28, 0.89) | 0.1021 | 0.50 (-0.56, 1.56) | 0.4830 |
| Water (Not Bottled) At Home | 0.07 (-0.17, 0.30) | 0.9614 | -0.16 (-0.54, 0.22) | 0.2470 | 0.60 (-1.07, 2.26) | 0.4913 |
